# Successful collaboration in dementia care from the perspectives of healthcare professionals and informal carers in Germany: results from a focus group study

**DOI:** 10.1186/s12913-015-0875-3

**Published:** 2015-05-28

**Authors:** Astrid Stephan, Ralph Möhler, Anna Renom-Guiteras, Gabriele Meyer

**Affiliations:** Faculty of Health, School of Nursing Science, Witten/Herdecke University, Stockumer Straße 12, 58453 Witten, Germany; Medical Faculty, Institute for Health and Nursing Science, Martin Luther University Halle-Wittenberg, Magdeburger Straße 8, 06112 Halle (Saale), Germany; Faculty of Health, Institute of General Practice and Family Medicine, Witten/Herdecke University, Alfred-Herrhausen-Straße 50, 58448 Witten, Germany

**Keywords:** Collaboration, Dementia care, Informal carers, Healthcare professionals

## Abstract

**Background:**

Informal carers of persons with dementia are in contact with numerous healthcare professionals (HCP) in a complex healthcare system. Successful collaboration between the parties involved appears to be essential for good dementia care. Thus, we investigated the perceptions of both HCP and informal carers regarding successful collaboration and sought to describe obstacles and facilitators.

**Methods:**

As part of the 7^th^ framework EU project RightTimePlaceCare, five focus groups were conducted with HCP and informal carers of persons with dementia in Germany (*n* = 30 participants/ time: Oct/Nov 2011). A supplementary secondary data analysis was performed, applying qualitative content analysis with open coding.

**Results:**

The derived categories were sorted into three overarching themes: *collaboration between HCP and informal carers, collaboration among HCP* and the impact of *resources and healthcare system*. HCP and informal carers largely agree on what facilitates or impedes successful collaboration between them. Making the initial contact appears to be a major challenge. While HCP expect to be contacted, informal carers hesitate to seek assistance, primarily due to inner barriers. Permanent contact person/institution, well-trained, empathetic HCP who can establish a trustful relationship are regarded as facilitating collaboration. The relational perspective is more clearly emphasised by HCP than by informal carers. This may be attributed to the absence of a permanent contact person in Germany.

Sufficient information relay, clear responsibilities, motivation and defined aims, and a personal relationship between professionals are mentioned as facilitators. External factors, such as rapid staff turnover, insufficient time resources and conditions specified by the health and long-term care system causing financial competition between providers, are described as general barriers to successful collaboration.

**Conclusions:**

HCP and informal carers had comparable perceptions of successful collaboration among them. The initial contact seems to be particularly challenging. Better strategies are urgently needed to facilitate the access to professional support. A permanent contact person (e.g., a case manager) might improve collaboration among all the parties involved, but this is not available regularly. Constraints created by the healthcare system may considerably hinder successful collaboration.

## Background

Persons with dementia suffer from progressive cognitive decline and deterioration in their capacity for living independently. Informal carers typically occupy a key position by taking responsibility for a person with dementia, particularly in the advanced stage of the disease [1, 2]. As there is currently no curative treatment available, strategies for coping with the disease and for supporting the families are particularly important [3]. Different types of support are required when the disease progresses, and persons with dementia and their families are in contact with a considerable number of healthcare professionals (HCP) working in different settings [3, 4]. Age-related multi-morbidity further increases the contacts with different disciplines and extends the complexity of interactions, often leading to the fragmentation of healthcare services [5, 6]. Thus both HCP and informal carers have to address numerous logistical and informational challenges. Therefore, successful collaboration among HCP and between HCP and informal carers appears to be crucial for good dementia care.

**Fig. 1:**
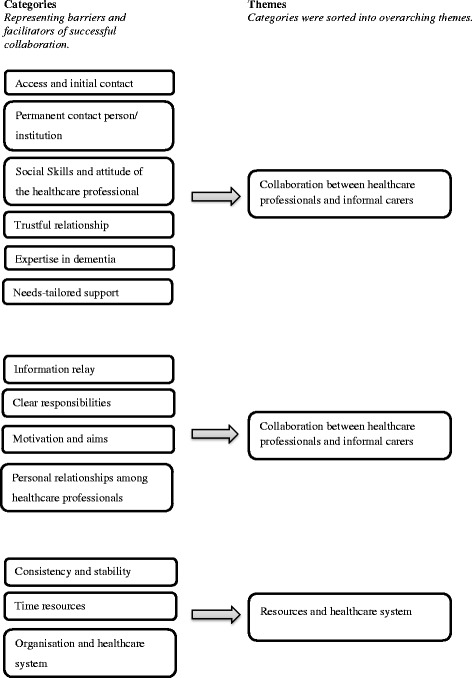
Overview of the analysis, derived categories and themes

Research focusing on inter-professional collaboration in dementia care is scarce. In general, concepts of collaborative care differ: while some concepts refer to collaboration within certain teams or between different types of professionals, others employ a broader perspective that focuses on collaboration between organisations and across settings [7]. Most studies address inter-professional collaboration between nurses and physicians [6]. Research suggests that successful collaboration between HCP depends on a number of aspects. While interactional factors between HCP are the most frequently investigated topic, the influence of the organisation and the healthcare system is rarely the focus of research [8].

From the perspectives of the informal carers who look after persons with dementia, interaction with both medical and care services appears to be a major challenge [9]. Informal carers experience a high level of burden [10, 11]. Moreover, they are typically the primary care manager and navigate an often non-transparent system of care providers [9, 12, 13]. An Australian study revealed that poor intersections between medical and non-medical or social services in care for older people hindered attempts to obtain information concerning suitable support services. Furthermore, multiple providers offering the same type of support were judged to be confusing, and carers criticised the unnecessary loss of time entailed by navigating complicated and partially overlapping services [9].

Creating a trustful relationship appears to be essential for successfully linking formal support and informal family care networks [9, 14, 15]. However, establishing relationships is considered a dynamic and complex process that requires negotiations over roles and potentially causes conflicts [13, 15]. Considering an informal carer as an equal partner instead of as a passive recipient of support is described as being an important precondition for a trustful relationship [9]. A meta-synthesis concerning the relationships among persons in need of care, informal carers and HCP in the home care context stresses that developing a type of a professional friendship can be considered a core characteristic of successful support at home [16]. Such a professional friendship requires a certain attitude among HCP, valuing the knowledge, habits and privacy of persons in need of care and their families [16].

### Dementia care in Germany

In contrast to many other European countries, Germany has no national dementia strategy; however, recently, initial attempts have been made in that direction [17]. The concept of *ageing in place* has been promoted: long-term care insurance provides partial financial compensation for informal carers or grants in-kind benefits, e.g., basic care at home [18]. Currently, the majority of persons with dementia are cared for at home by informal carers [18], whereas home care services are involved in approximately one-third of caregiving situations [19]. However, the number of arrangements involving both formal and informal care is expected to increase [15]. A variety of services is available, and the care system is highly fragmented. According to market principles, a mix of public, private or charitable care services is available, and the local authorities’ steering function is limited [20].

The diversity is further increased by the federalism in Germany, which attributes a high level of freedom with regard to the design of services to the federal states and the counties but also to individual municipalities.

A lack of care coordination further contributes to a non-transparent and complex system [20]. In recent years, so-called *dementia networks* have been widely introduced, with the aim of cross-linking different care providers to improve and better coordinate local dementia care structures [21]. However, successful collaboration between HCP and informal carers of persons with dementia and among HCP themselves appears to remain a major challenge for the German dementia care system. We therefore sought to explore the views and experiences of involved parties (HCP and informal carers) with regard to successful collaboration in dementia care.

## Methods

### Research aim

We sought to explore the views of HCP and informal carers of persons with dementia with regard to successful collaboration (1) between HCP and informal carers and (2) among HCP themselves and to describe the factors that impede or facilitate collaboration.

### Design

As part of the 7^th^ framework European project RightTimePlaceCare (RTPC) [22, 23], a focus group study was conducted in Germany. Focus groups are characterised by active discussion among the participants, guided by a moderator [24]. The method provides insights into individual experiences and enhances understandings of the factors that influence behaviour or motivations [25, 26].

### Group composition

Homogeneous groups are generally recommended because individuals may feel more comfortable offering their opinion in such a context [24, 26]. We expected that HCP and informal carers of persons with dementia have different perspectives or even different levels of power in relation to the topic [13] and we therefore decided to conduct separate focus groups.

### Participants

For the first type of focus group, HCP working in different dementia care settings were approached. The group was to include various types of HCP involved in dementia care, such as nurses, physicians or social workers. As an important inclusion criterion, the HCP had to work in a good-practice working environment with regard to the collaboration between all parties involved.

For the second type of focus group, informal carers of persons with dementia in different stages of the disease were approached in order to draw on a comprehensive range of experiences. Informal caregivers were defined as a relative, friend or neighbour mainly involved in caring for the person with dementia.

We initially intended to include the views of persons with dementia. Because this was expected to be difficult, we also invited *proxies* (i.e., counsellors, leaders of support groups) who were able to voice the opinions of persons with dementia.

### Recruitment

Participants were purposively recruited. In order to find eligible HCP, the German Alzheimer’s Society was contacted to identify HCP working in a good-practice working environment. Furthermore, municipalities or pilot projects were selected which the research team knew to be good-practice examples of collaboration. Written information concerning the project, including an invitation to participate, was sent to the respective institutions, networks, pilot projects or municipalities. The German Alzheimer’s Society recommended four good-practice examples, two of which sent a participant (from southern [Bavaria] and northern [Schleswig-Holstein] Germany). Six known good-practice examples (western Germany, North Rhine-Westphalia) were identified by the research team and colleagues at Witten/Herdecke University, four of which sent one or two participants.

Informal carers were contacted via support groups or counselling agencies. Furthermore, existing contacts with institutions and care providers established within the RTPC project were used (e.g., nursing home care organisations, day care centres). Potential participants were approached by contact persons within the institutions, and written information and invitations were provided. In the event that they were interested in participating, they were contacted by a member of the research team. In total, 9 out of 14 eligible persons participated (Groups C and E). Only one person with dementia could be included, but three proxies participated. The invited proxies also had some type of caregiving experience and thus primarily reported from the perspective of an informal carer. The participants did not know one another beforehand, except for the woman with dementia, who participated in the company of her daughter. Additionally, a focus group was conducted with a support group of informal carers that had existed for several years (Group D).

### Data collection

The focus groups were held in October/November 2011, either at the university or appropriate locations proposed by the participants. The discussions were tape-recorded and transcribed verbatim. A moderator (AS or GM) and an observer (GM, ARG or trained study assistants) conducted the focus groups. A common questioning route ensured a certain degree of consistency across all the focus groups and across the countries [23]. The interviewer followed the questioning route closely, with the intention of not influencing the discussion. The observer remained silent and took notes. Positive experiences or good-practice examples and their features were solicited and negative experiences were addressed to obtain contrasting examples. A simple opening question was employed to encourage involvement (*Please describe the HCP involved in caregiving/you are collaborating with*). The core of the focus groups was an open discussion initiated by asking a few key questions (*When do services work at their best? Tell us about situations when a service works really well in terms of collaboration? What characterises the service at those times?*). The focus groups concluded with a closing question to consolidate the discussion (*Having the opportunity to tell the government - what would you like to see improved in relation to collaboration?*).

### Data analysis

The presented analysis can be considered a secondary analysis, i.e., a supplementary analysis [27]. The primary analysis was performed as part of the RTPC project, following a predefined coding scheme that was applied in all countries. The condensed findings from eight European countries were merged and needed to be decontextualized and abstracted [23]. The present study extends this analysis and seeks to be more specific by focusing on the barriers to and facilitators of good collaboration, with specific consideration of the German context. We followed an interpretive paradigm [24] and performed an inductive content analysis applying an open coding procedure [28, 29]. The data analysis was supported by the qualitative data analysis software MAXQDAplus version 11 (VERBI GmbH, Berlin, Germany).

### Description of the analysis procedure

We followed the recommendations given in the literature [30] and agreed with the assumption that dialogue among researchers is highly valuable within an open coding procedure [29]. Thus, two researchers performed the analysis, applying a predefined procedure. Categories were derived separately for the HCP and informal carer focus groups. The first transcript of each type of focus group was analysed independently by two researchers (AS, RM [RM was not involved in the study]). The transcripts were read several times and meaning units, such as words, sentences or paragraphs, were identified to condense the content. As a first attempt to abstract and interpret the content, the meaning units (sentences or paragraphs) were labelled with codes. Based on these codes, categories were developed that were considered to be the manifest content of the transcript [29].

Both researchers discussed and challenged their independently derived categories and coding decisions critically in personal meetings (or via video conference) and thus developed a joint system of categories. The first transcripts from each type of focus group (HCP and informal carers) were then re-checked to confirm these categories. Subsequently, all of the material was analysed by one researcher (AS), and was at the same time constantly checked to see whether the interpretations and the description of the categories are corroborated by the data of the other interviews; modifications were possible throughout the analysis. Discussions between AS und RM were held during the analysis whenever necessary. As a final step, the system of categories was jointly reviewed by AS and RM and the categories were sorted into global themes. The other members of the research group checked the categories for plausibility (ARG and GM) [29].

### Trustworthiness

According to the requirements of qualitative research, trustworthiness is defined as credibility, dependability and transferability [31]. Measures were taken to enhance credibility, i.e., by involving two researchers in the analysis [29], one of whom was not involved in the data collection or primary analysis. As a final step, the categories were critically reviewed by the two researchers and discussed with the research team to ensure its plausibility and understandability. We did not perform member checks because the period of time (three years) between conducting the focus groups and performing the secondary analysis was considered too long (due to possible recall bias and unavailability of former participants). To further ensure translational credibility, a native speaker of British English (with longstanding experience as an assistant in nursing and health research studies) was involved in the translation of the system of categories and quotations into English. Moreover, a wide range of different participants was included: the informal carers covered various caregiving experiences; the HCP had diverse professional backgrounds and worked in different working environments in different parts of Germany. Dependability was ensured by following a questioning route that the cooperating research group piloted in Sweden [23]. To ensure transferability, we sought to describe the data collection, analysis procedures and the results in as much detail as possible.

### Ethical considerations

Ethical approval was granted by the German Society for Nursing Science (August 2011) and the study was carried out in compliance with the Helsinki Declaration (http://www.wma.net/en/30publications/10policies/b3/index.html). All participants gave their written informed consent prior to the focus groups.

## Results

### Participants

Two focus groups with HCP (*n* = 13 in total) and three focus groups with informal carers (*n* = 17 in total) were conducted (Table 1 and 2).

**Table 1:** Characteristics of the healthcare professionals

Group	Type	Participants	Gender	Age (years)	Working environment	Interview duration
A (*n* = 6)	Arranged group	2 nurses, 1 social worker, 1 psychologist, 1 geriatrician, 1 member of a municipality (staff position, responsible designing for dementia-friendly municipality)	3 female, 3 male	32-51	day care centre, providers of nursing homes including day & respite care, acute geriatric unit, municipality, counselling agency	85 min
B (*n* = 7)	Natural group (steering group of a dementia network)	3 nurses (further education in management or psychology), 2 social workers, 1 social pedagogue, 1 psychiatrist	7 female	37-62	Community care services (on an organisational level), counselling agency, outpatient clinic	74 min

**Table 2:** Characteristics of the informal carers

Group	Type	Participants	Gender	Age (years)	Living situation of the person with dementia	Interview duration
C (*n* = 6)	Arranged group	2 informal carers 1 PwD & 3 proxies	5 female, 1 male	57-79	2 at home^a^	134 min
D (*n* = 7)	Natural group (support group for carers)	7 informal carers	3 female, 4 male	64-90	4 at home, 1 nursing home, 2 PwD deceased	70 min
E (*n* = 4)	Arranged group	4 informal carers	4 female	47-64	2 nursing home, 2 deceased	92 min

^a^PwD participated together with her daughter

### Group dynamics

The contributions of the HCP were quite balanced. However, within Group B (the steering group of the local dementia network), one member remained silent (nurse), and the other participants often sought eye contact with the leader of the steering group when they contributed. The silent participant was invited to contribute by seeking eye contact and inviting gestures (such as nodding) but was not directly addressed in order to avoid urging her to speak, which could have led to unreliable or evasive answers [26]. The discussions between the informal carers were also balanced: all the participants joined in the discussion except for the person with dementia (who offered very few statements).

### Findings

A high level of agreement between HCP and informal carers was revealed, although the categories for both types of focus groups were derived separately. When reviewing the categories, three overarching themes emerged: *collaboration between HCP and informal carers*, *collaboration among HCP* and the impact of *resources and healthcare system.*

### Collaboration between HCP and informal carers

#### Access and initial contact

HCP and informal carers both describe *access and first contact* as a major challenge for good collaboration. The HCP stress the difficulties of making contact with the informal carers. They believe that they should wait to be contacted and regard the informal carers as responsible for seeking assistance. Thus, the HCP develop strategies to facilitate being contacted by informal carers (e.g., being recognisable to ensure that families can easily locate a care provider or a service). Furthermore, they feel that they have to be connected with other HCP and services within their district to allow them to refer the consulting families quickly to an appropriate contact person/institution.

*“I often think they could have known something earlier or found some support. Then they wouldn’t have been so burnt out when they got here.”* (Group B, nurse/paragraph 22)

Achieving early contact with informal carers is consistently described as a facilitator for good collaboration.

*“As I see it, the earlier the better and easier it is. Early and lasting support.”* (Group B, psychologist/paragraph 129)

The HCP describe several reasons for late contact, primarily on the part of the informal carer, such as a strong sense of duty regarding the person with dementia, the expected costs of professional support or a failure to recognise that support might be necessary.

*“After the door opens things get easier, I think, but before that, I find it really very difficult. […] when the people open the door to you*, *then the first step is taken. But before! How often have I seen that people resist for ever so long and say: ‘No, no, we can manage, we don’t need help.’ Until it all escalates.”* (Group A, nurse/paragraph 40)

HCP are often contacted for the first time during a crisis or even when the caregiving system has collapsed, which is perceived as a barrier to good collaboration. The expectations of the informal carers regarding available support are described as high within a crisis, whereas the HCP’s scope of action is limited. HCP are unable to create a stable network of services on short notice (e.g., day care, home care and volunteer support), which may often lead to an unexpected admission to a long-term care institution.

The informal carers also see the *access and first contact* as a major challenge. They report that they have difficulties in contacting HCP, primarily due to their own perceived inner barriers or uncertainties regarding important decisions to be made, such as establishing the diagnosis, using professional community support or moving to a long-term care institution. This means that substantial time may elapse before informal carers actively seek professional support.

*“Because at first you just don’t want to accept it. I think you just want to do everything on your own because you are, you must … surely that’s how it is? I can do that!”* (Group D, daughter/paragraph 97)

Informal carers may realise retrospectively that more proactive support or counselling was needed because they were unable to take the first step or to make a care decision.

*“I didn’t mention it because I knew what would happen. And that’s exactly what I didn’t want (note: moving into a nursing home). I think I should have had more attention there […] I should have had someone from outside tell me: This can’t go on any longer. Perhaps I might have resisted even then […]. Looking back, I can see that the situation was clear, either I’d have collapsed or he would have. Then he did collapse and had to go into hospital and everything was clear after that. But I, well, actually I should have had some help, but I didn’t do anything about it.”* (Group D, spouse/paragraph 329–333)

#### Permanent contact person or institution

Both HCP and informal carers consider a permanent contact person or institution to be essential for good collaboration. HCP stress that one person should be responsible throughout the course of the disease to guide the informal carer, ensure information supply and manage the services involved.

*“Actually, I believe you need someone who is responsible for everything and who can pass on all the information and tell you what help is available. From healthcare power of attorney to the needs assessment and up to parenteral nutrition at the end: yes or no? You need someone who can guide you through all this, for me that would really be optimal care.”* (Group B, nurse/paragraph 49)

When informal carers begin to seek professional help, they must navigate a highly complex system involving different providers and numerous counselling offers, especially in larger cities. They appear to be predominantly responsible for obtaining information on available services, for organising care and transferring information among various HCP.

*“It’s like collecting little bricks and building a house.”* (Group E, niece/paragraph 51)

All three group discussions with informal carers reveal that a permanent contact person would be a support and guide for the informal carers. Occasionally, informal carers may locate such a person or institution that was available only during a certain period, or in some cases throughout the course of dementia. The Alzheimer’s Society, where one of the focus groups was held, provides services which may involve such continuous counselling and support. However, other examples of a permanent contact person exist, as the following example illustrates, in which a Dementia Café served as a stable contact point throughout the course of dementia.

*“[…] for four years I kept on going to this Dementia Café that was organised by an old-age therapist who used to work on a ward in the nursing home. Through all the years, this thread guided me up to the end; I had personal contacts as well as professional contacts. Then my husband came on the same ward where this old age therapist had worked, and yet again I had close support until his death.”* (Group E, spouse/paragraph 63)

#### Social skills and attitude of the HCP

HCP emphasise that certain skills and attitudes are essential for good collaboration with informal carers. Features such as openness, friendliness, being approachable and being proactive in seeking and maintaining contact with informal carers are described as facilitators.

“*And that is a great quality, I think: a personal relationship. To make a contribution and give someone, the person you are talking to, the feeling that what has happened is authentic.”* (Group A, social worker/paragraph 43)

This is confirmed by informal carers, who expect the HCP to be patient, approachable and honest. Honesty also includes describing the limitations of professional support instead of making promises that cannot be fulfilled. Informal carers particularly expect the HCP to value their knowledge and experiences in caring for a person with dementia because they contribute significantly to need-driven care. When the advice of the informal carers is not considered, this is regarded as a major barrier to good collaboration. Furthermore, the informal carers emphasise that HCP should respect the person with dementia, and this is regarded as a facilitator of collaboration:

“*A prerequisite for working well together, it is so important to show an understanding attitude. That means that the individuals, the people in need of care, are really the centre point. And that people aren’t ruled over. I’ve experienced that so many times[…]*” (Group A, daughter and volunteer/paragraph 62)

#### Trustful relationship

HCP emphasise the need for a *trustful relationship* as an essential precondition for good collaboration, although this aspect is not so evident in the groups of informal carers. To develop a trustful relationship, informal carers must feel confident that the HCP would act in their interest and according to the needs of the person with dementia. HCP describe certain features, such as the abovementioned social skills and attitudes, and a continuous contact person as important preconditions for establishing trustful relationships.

“*Well, regarding the relatives I’d say, it’s the approach, not only do you have to find an opening, but you have to keep it open […] you can’t set up a trustful relationship just once and for all, you have to keep working on it*.“(Group A, nurse/paragraph 110).

#### Expertise in dementia

Informal carers stress *expertise in dementia* as a precondition for good collaboration, while this is rarely mentioned by the HCP. From the perspective of informal carers, HCP require an understanding of dementia, but such understanding often seems to be insufficient, particularly within the hospital setting. Furthermore, only specially trained individuals should perform counselling:

*“Competent counsellors, who have been properly trained in geriatrics and dementia, can fulfil this advising function. Well, I find it important to know there’s someone you can talk with, someone who understands!”* (Group E, daughter/paragraph 352)

#### Needs-tailored support

Informal carers note that support should be tailored to their needs, an issue which is confirmed by the HCP. Inflexible structures and services are perceived as negative and are considered to impede good collaboration. An example of such inflexibility is a home care service that offers assistance only during a certain period of time each day.

*“If she were bedridden” (Spouse 2 “Yes, that’s different!”) “but with my wife I just don’t know how that would work, if they (comment: the nurses) come at a fixed time. And I don’t know whether my wife wants it. Sometimes I need an hour until I can get her washed […].”* (Group D, two spouses/paragraph 165)

#### Collaboration among HCP

Informal carers only indirectly address collaboration among HCP, e.g., when collaboration between HCP is insufficient and they have to be the primary care manager. In contrast, the HCP discuss inter-professional collaboration comprehensively, and it emerged that collaboration occurs at three levels: Within organisations, across organisations and within so-called *dementia networks*.

#### Information relay

An unimpeded relay of information is described as a precondition for collaboration between HCP. With insufficient information, the basis for collaboration is lacking (described particularly for transitions between hospital and community care). An adequate relay and use of information would contribute to more continuous care.

*Nurse: Yes, well, that you know more about each other so that every new provider doesn’t have to start at zero or, if you like, at number one. But everybody starts again from the beginning and doesn’t make use of the information in the earlier reports, or the knowledge available*, *everyone just gets going and muddles along and gives up if they’re at a loss. That’s putting it a bit bluntly, I know.”*

*Social pedagogue: “Keeping this logbook, huh? For instance.”*

*Nurse: “Lasting support, so everybody is informed […].”* (Group B, social pedagogue and nurse/paragraph 149–150)

#### Clear responsibilities

Clear responsibilities are especially stressed as an important precondition for good collaboration within dementia networks consisting of different HCP. Here, responsibilities are not per se determined by the professional background or the respective position of the HCP. New responsibilities have to be established, and leadership becomes an important feature of good collaboration in dementia networks.

*“I think that’s always the important thing with the networks, that somehow there’s someone there who holds it all together.”* (Group B, social worker/paragraph 27)

#### Motivation and aims

Motivation and defined aims are necessary when HCP collaborate with one another. HCP may realise, for example, that collaboration might ultimately lead to a reduced workload or that joining a dementia network extends one’s expertise and thus may promote good professional collaboration. Moreover, need-tailored support for persons with dementia and their families represents a strong motivation for good collaboration. This refers also to collaboration across organisations, when HCP attempt to determine the most appropriate support offered by other providers.

*“I could put it another way: If in the end the individual situation of the patient was the crucial point, no matter which provider is involved, or what budget is available. Or the family, if THAT was the starting point of all the deliberations, if that was the motive, then – then that would be a dream.”* (Group A, social worker/paragraph 120)

Financial and economic interests and the aims of the care providers are described as important barriers to good collaboration across organisations and within dementia networks. Good collaboration may be best established by defining common aims, including economic components.

*“They are commercial enterprises (note: the providers), you just have to accept that.” (Psychologist: “It’s politically intended.”*) *“Win*-*win, you’ve got to reach a win*-*win or convey that you can show how someone can profit from it […].”* (Group A, psychologist and member of a municipality/paragraph 27–29).

#### Personal relationships among HCP

Moreover, regarding inter-professional collaboration, a personal relationship is considered an important facilitator of good collaboration, contributing to mutual understanding and improved communication structures:

*“I have been really lucky that in the meantime we’ve got to know each other privately. That has really made a huge difference, although it sounds strange, but this private contact has opened our eyes to one another.”* (Group A, nurse/paragraph 60)

#### Resources and healthcare system

##### Consistency and Stability

Consistency and stability appear to be important features of good collaboration among HCP and refer to different levels and ranges of actions. Regular meetings between HCP within a given institution and across organisations are described as a strong collaboration facilitator. Moreover, HCP often mention that “*long*-*term relationships or structures”* are an important facilitator of good collaboration, so the individual carers ought to remain in a given position for a certain period of time to allow such relationships or structures to develop.

“*The other thing is, well, long*-*term relationships. The people in leading positions have all been here for over 15 years, and a lot of the nurses, too. The better you know each other, of course, the easier it is to shape or form this particular culture. And that’s the problem in many institutions, where especially managers’ heads are always rolling, especially nursing directors (the others nod in agreement*)*; a communication structure is very difficult to develop.”* (Group A, psychologist/paragraph 21)

##### *Time resources*

Time restrictions and a lack of financial compensation for collaborative work are consistently reported as major barriers, both among HCP and between HCP and informal carers.

“*I wish there was a market value, a cash value for the ‘care*-*manager’. And this job gets the acknowledgement it deserves, not only morally but financially as well, then a lot would change.”* (Group A, psychologist/paragraph 121)

Furthermore, informal carers perceive a lack of time as a barrier to good collaboration. When a HCP is responsible for too many individuals, it is obvious that the time for suitable collaboration with informal carers is lacking, as the following example of a nursing home situation demonstrates:

“*[…] I think it was a problem even then and these nurses, they were all very, very nice and there was a nurse*, *who was responsible for him and I could ask whatever I wanted. The problem was that after two minutes she said: ‘I have to get on*, *otherwise I can’t get my work done’ […].”* (Group E, spouse/paragraph 68)

##### *Organisation and healthcare system*

The HCP identify major barriers to good collaboration in the competitive health and long-term care systems in Germany. The structures of these systems often mean that financial interests become paramount among the care providers or HCP working in private practices, which may prevent the realisation of need-tailored support for persons with dementia and their families. The HCP particularly criticise the lack of a legal foundation for home care services to adopt a preventive approach and be proactive in seeking early contact with persons with dementia and their families. This is described as an important barrier to establishing a timely and trustful relationship.

“*If we could open the door by giving out medication because there are no psychiatric billing codes. Well*, *there are*, *but you don*’*t get them. You have to fill out such a lot[…]. The chances for operating as a professional are missing. That*’*s a huge failure on the part of the health insurance system […].”* (Group A, psychologist/paragraph 63)

## Discussion

Overall, three main themes emerged: *collaboration between HCP and informal carers*, *collaboration among HCP* and the impact of *resources and healthcare system*. The results revealed that HCP and informal carers largely agree on what facilitates or impedes good collaboration between them.

Both regard access to and the first contact with HCP as a major challenge. The HCP emphasise that the current system offers few possibilities for seeking early contact with persons with dementia and their families and adopting a preventive approach. Thus, the HCP wait to be contacted, while informal carers appear to experience considerable difficulties in overcoming inner barriers and contacting HCP. They may hence require a more proactive support. This appears to create a dilemma, in that HCP regard early contact as a facilitator of good collaboration, whereas waiting until a crisis emerges appears to be a further barrier to good collaboration. The expectations of the informal carers during a crisis are described as high, whereas the HCP’s scope of action is restricted. Timely contact would enable HCP to plan in advance and in close collaboration with the informal carer and the person with dementia. Social networks could be strengthened and need-tailored services could be established in order to prevent a crisis or to deal appropriately with critical moments.

That informal carers often approach HCP quite late may be partially explained by the concept of ambiguous gain [32]. While, on the one hand, professional support may relieve informal carers of physical stress, on the other hand, it may also have a negative impact, e.g., informal carers may be unable to reconcile the professional system and their real world, or the use of professional support may be regarded as personal failure or a loss of mastery. Our findings provide indications in this direction, but further research on the concept of ambiguous gain is needed in order to understand the intrinsic barriers informal carers face in using professional support (or lack thereof). Interestingly, this issue did not emerge from the cross-national analysis combining the findings from the other countries participating in the RTPC project [23].

HCP and informal carers agree that a single contact person/institution is an important facilitator of good collaboration. A continuous contact person (e.g., a case manager) is presumed to facilitate access to dementia support [9, 12] and is considered good practice in social care for persons with severe and complex care needs [33]. The findings of our international focus group study confirm that a continuous contact person is crucial in all participating countries [23]. Moreover, a systematic review suggests that informal caregivers could directly benefit from improved collaboration with HCP, as this was sufficient to relieve their burden [34]. Thus, informal carers should clearly be relieved of administrative and management tasks, which our findings indicate were considerably burdensome.

Our findings further reveal that social skills and a certain attitude are important characteristics of HCP that generate good collaboration with informal carers and among HCP. Skills and attitudes are considered an essential aspect of dementia nursing competences, but these are scarcely described from a multi-professional perspective [35]. While the literature treats the formation of a trusting relationship as a major topic for informal carers [9, 16], this aspect was more clearly emphasised by the HCP than by the informal carers in our study. The absence of a continuous contact person for informal carers in Germany [36] may indicate that this relationship is less valued from the carers’ perspective. The informal carers in our study were more likely to emphasise the importance of dementia-specific knowledge and tailored care that suits their situation, which is in line with prior research stressing the positive impact of responsive services [9].

In our study, informal carers devoted little attention to inter-professional collaboration among HCP, whereas HCP discussed inter-professional collaboration intensively. Three levels of collaboration were important for these HCP, demonstrating the complexity of collaborative work: collaboration within an institution, across institutions and within formally initiated dementia networks. This may be partially influenced by the fact that the participants primarily held management or comparable leading positions and participated in dementia networks.

In our study, HCP devoted some discussion to basic aspects of collaboration, such as guaranteed information supply, revealing that available information is apparently not always employed properly. Research suggests that information supply is a necessary but not sufficient precondition for good collaboration [37]. Instead, coordination has been identified as a key feature of inter-professional and inter-organisational collaboration [7, 8, 38]. Our findings support the importance of coordination in the form of a contact person serving as a key contact for families and other HCP.

Further important aspects are the motivation and aims of HCP. While sharing common aims serves as a facilitator (such as need-tailored support for a certain person with dementia), the financial interests of care providers may impede good collaboration among HCP. Thus, discussing aims and motivations appears to be important when multiple HCP have to collaborate, especially within inter-organisational collaborations or dementia networks. This is in line with the literature, which indicates that the willingness to collaborate is influenced by expected benefits and by sharing common objectives [8, 39]. However, neither the literature [39] nor our findings provide further information on how such common aims might be best established. The consideration of economic aspects and the market-driven healthcare system appears to be particularly important for Germany, especially with respect to fostering inter-organisational collaborations and dementia networks, which are being increasingly established.

Moreover, the HCP stressed the importance of having personal, trusting relationships with other HCP, which is supported by research and conceptual studies, indicating that collaboration needs to be considered as a human process [6] and that aspects such as trust, mutual respect and positive interpersonal relations are features of good collaboration [8, 38, 39]. Thus, offering sufficient opportunities for HCP to make personal contacts may influence collaboration positively. The attention devoted to the organisational and systemic determinants of good collaboration has been particularly limited in empirical studies [8]. Our findings suggest that certain external factors may serve as facilitators of good collaboration, such as low staff turnover and sufficient time to develop and maintain relationships, whereas the market-driven healthcare system is perceived as a barrier to good collaboration, giving rise to competition between different care providers.

### Strengths and limitations

The variety of perspectives is certainly an important strength of our study. Contrary to the majority of empirical studies investigating inter-professional collaboration, we did not focus exclusively on nurse-physician relationships [8] but included different types of HCP involved in dementia care throughout the course of the disease. Moreover, the HCP held different positions in areas known as good practice examples of collaborative practice and came from different parts of Germany. The informal carers had a broad range of experience in caregiving and in collaboration with HCP.

A potential limitation is that we were unable to include general practitioners, who are often considered important contact persons for persons with dementia and their families in Germany [36]. Another limitation might be that, despite recruitment efforts, we identified only one person with dementia willing to participate together with her daughter. Including persons with dementia in qualitative research remains a challenge [40]. Better recruitment strategies and, possibly, additional time for recruitment are necessary to include a sufficient number of persons with dementia in focus groups. However, the overall schedule of the European project determined the data collection period. Although it remains uncertain whether data saturation was achieved, the high level of agreement among participants, including the international analysis [23], suggests that a comprehensive description of successful collaboration covering various perspectives was realised.

It seems reasonable that successful collaboration depends on a variety of barriers and facilitators that are probably inter-related and have effects on different levels. However, this issue remains open since qualitative content analysis primarily identifies the content in a descriptive way and does not per se provide techniques to investigate the relationship between concepts and categories [30].

The structured analysis procedure and involvement of a team of researchers can be considered a strength. Two researchers performed the analysis, one of whom was not involved in the data collection or in the initial analysis and who was hence unbiased and open. Translational credibility was ensured by consulting a native speaker.

## Conclusion

HCP and informal carers have comparable perceptions of what constitutes good collaboration. The initial access and first contact are perceived to be particularly challenging by both groups. While HCP describe obstacles in approaching persons with dementia and their informal carers (primarily attributed to the healthcare system), informal carers emphasise difficulties in taking the first step and contacting HCP (attributed to internal barriers). Better strategies are urgently needed, seeking to enhance the first contact between informal carers and HCP and facilitating the access to professional support. Moreover, a continuous contact person (e.g., a case manager) for informal carers and HCP is not generally available in Germany, and this appears to be an important barrier to good collaboration, both between HCP and informal carers and among HCP. As the literature notes, HCP cannot create inter-professional collaboration alone [8]. Our findings suggest that constraints determined by the health and long-term care systems may considerably hinder good collaboration, even within dementia networks or working environments exhibiting good collaborative practice.

### “What this paper adds” box

#### What is already known on this subject?

Informal carers of persons with dementia must navigate a complex and fragmented healthcare system. Successful collaboration between the parties involved appears to be essential for realising good practices in dementia care. The majority of persons with dementia in Germany are cared for at home, and the involvement of professional care is expected to increase. According to market principles, a mix of public, private and charitable services is available in Germany. Thus, successful collaboration among HCP and between HCP and informal carers is a major challenge for the German dementia care system.

#### What this study adds

We explored the views of HCP and informal carers of persons with dementia regarding successful collaboration. HCP and informal carers largely agree on what facilitates or impedes good collaboration. Both consider a single, continuous contact person/institution as an important facilitator of successful collaboration. Making the initial contact appears to be a major challenge in Germany. While HCP wait to be contacted, informal carers describe difficulties in seeking assistance and often wait until a crisis emerges. Further obstacles are attributed to external factors such as staff turnover, insufficient time resources and conditions determined by the healthcare system that generate financial competition between care providers.

## Abbreviation

HCP: Healthcare professionals
